# Progress in Medicine

**Published:** 1900-05-12

**Authors:** 


### Progress in Medicine.

### RHEUMATISM AND GOUT.

(*Continued from page* 85).

**Gonorrhœal Rheumatism.**—T. O'Connor[13] continues to recommend early incision with irrigation and drainage. He now gives the notes of ten cases, in nine of which he obtained a complete cure by this method. At the time of writing no instance of sepsis or relapse had occurred. Christopher Heath[14] lays down that the disease is a mild form of pyæmia due to the purulent secretion within the urethra, and a special feature of it is that it first attacks a single joint in a young adult. He gives five grains of quinine every six hours, avoids splints, and merely applies belladonna and glycerine to the affected joint, and does not treat the urethral discharge at all. Under this remarkable method he claims to cure his patients in three weeks. Moynihan[15] recognises four forms of gonorrhœal arthritis—serous, sero-fibrinous, purulent, and phlegmonous—and points out, as a feature of the disease, that the complaints made by the patients are frequently out of all proportion to the severity of the local inflammation, and that this may sometimes aid us in diagnosis. He denies that the disease usually appears first in a single joint. First of all flying pains are felt in several joints and are accompanied with some difficulty of movement. After this a severe attack may settle on a single joint. If the gonorrhœa has reached a late stage there are generally two or more articulations affected at once with different forms of the disease. He quotes some German statistics, showing, however, that mono-articular cases are twice as frequent as polyarticular ones. As to treatment, he prefers to give mercury, or iodide in large doses, and for the joint he advises complete rest at first, to be followed by passive movement at an early stage. Painting with iodine, or in severe cases immediate incision may be desirable, but the most common danger is that of ankylosis.

**Purpura.**—The use of gelatin injections in aneurisms and for various surgical procedures has been recommended by many writers recently. Arcangeli[16] has employed it successfully in purpura hæmorrhagica. A 2 per cent. solution in normal saline solution was injected into the subcutaneous abdominal tissues. The patients were girls, aged 13 and 10 years respectively. The elder received an injection of 20 c.c. on two successive days, and the younger others of 15 c.c. and 10 c.c. Nichols,[17] like others, has used this treatment for hæmophilia, but he alone seems to have employed it externally, for he poured melted sterile gelatine into a troublesome wound from which bleeding had gone on persistently. After packing it also with iodoform gauze no more hæmorrhage took place, and healing by granulation came in the normal course.

**Gout.**—Kionka[18] believes that human and avian gout are identical, and the manifestations can be caused in either case by an excessive meat diet. In fowls the excretion of uric acid can be reduced by a half if calcium salts are given in the food, and certain analyses show a reduction of the formation and excretion of uric acid in men to whom calcium salts have been long administered. Though the matter is not yet fully proved, it is significant that all mineral waters used for gout show a large percentage of calcium salts. The origin of uric acid from nuclein has been hotly discussed. Horbaczewski thought that it arose from the nuclein of broken-down leucocytes, others derived it from the nuclein contained in food, while a third theory traced it to metabolic changes in the tissues of the body. F. S. Jerome[19] shows evidence in favour of its origin from nuclein, nucleo-proteids, and one form of alloxuric base. Thus, he found an increase after feeding on fishes' roe, Liebig's extract, or pure nuclein. He would regard the uric acid excreted after a flesh meal, before complete digestion, as due to hypoxanthin and other substances in the meat, and not from preformed nuclein, though he finds that the addition of nuclein to the diet is followed by an increase of uric acid at a later period. C. C. Douglas[20] reports a large number of simultaneous estimates of uric acid and leucocytes, and finds that there is a discrepancy between the two. Though an increase in both occurs in certain circumstances, there are many cases in which no such relation exists. He therefore does not believe that the source of uric acid is normally in the nuclein of the leucocytes, and refers also to the objection which has been made on the ground that no increase of phosphoric acid accompanies that of uric acid. If uric acid were derived from leucocytes, any breaking up of the latter would also supply phosphoric acid. Still, he confesses that nuclein containing foods may increase the uric acid excreted, and, therefore, he warns us in feeding the gouty not to give sweetbread and similar substances, or vegetables rich in proteids.

It is interesting to find an author exonerating uric acid altogether. A. C. Croftan[21] argues that the evils usually attributed to it are really due to the alloxuric bases, and that uric acid is quite harmless, except for its tendency to form concretions. In diseases where its excretion is subnormal, the cause is that less is formed, not more retained, though in affections of the gouty type there is a great increase of alloxuric substances. He claims that the former defective methods of estimating uric acid invalidate the statements of Garrod and others. He declares that uric acid can be found in normal blood, that its excretion is not diminished in Bright's disease until the final stage, nor in gout, but in the latter case there is an actual excess of the alloxuric bodies. In fact, the formation of uric acid is protective, and the normal mode of getting rid of broken-down nuclein. By injections of hypoxanthin and similar substances he has succeeded in producing kidney lesions similar to those seen in gout and lead poisoning with albuminuria, while uric acid injected for three months caused no kidney lesions whatever. The practical deductions he makes are—in early stages of gout we should avoid everything which causes a leucocytosis, and in later stages we should do what is possible to increase oxidation. Thus, in acute gout, he advises inhalations of oxygen.

Another investigation of the whole subject has been attempted by Chalmers Watson,[22] who also throws doubt upon Garrod's theories. He thinks that the observations of M. Levy and those he has himself carried out prove that (1) the alkalinity of the blood is not diminished during an attack of gout; (2) nor is the excretion of uric acid diminished, (3) while the amount of uric acid in the blood is no greater than in the period between attacks. Of course, if these results are accepted the whole theory as to the uric acid being the cause of the paroxysm is disproved. He is inclined to minimise the importance of uric acid in the production of the disease, and holds that more study is needed of the relations of uric acid to other similar substances, and of the characters of the blood. Thus, in leucæmia we have a marked excess of uric acid in the blood, and yet none of the clinical symptoms of gout. He regards as unproven also the view that uric acid exists in the blood in the form of a quadriurate, though this has been generally accepted by most writers. The effect of a diet rich in nuclein on the excretion of uric acid has been estimated in a contradictory manner by various writers, and the author's own experiments do not appear to clear up the point. Similarly, with regard to the relation between the uric and phosphoric acids excreted, he expresses the view that much more observation is needed before definite conclusions can be reached. Indeed, the whole paper is a plea for fresh investigation of points which had been thought to be more or less settled.

[13] Lancet, Dec. 9. [14] Lancet, Nov. 25. [15] Lancet, Nov. 18. [16] New York Med. Jour., Dec. 16. [17] Brit. Med. Jour., Dec. 23. [18] Med. Rec., Nov. 11. [19] Brit. Med. Jour., Jan. 6. [20] Edin. Med. Jour., Jan. [21] Brit. Physic., Mar. 15. [22] Brit. Med. Jour., Jan. 6.

